# The inverse association between cancer history and incident cognitive impairment: Addressing attrition bias

**DOI:** 10.1002/alz.14268

**Published:** 2024-09-26

**Authors:** Michelle Shardell, Alan M. Rathbun, Ann Gruber‐Baldini, Alice S. Ryan, Jack Guralnik, Dimitrios Kapogiannis, Eleanor M. Simonsick

**Affiliations:** ^1^ Institute for Genome Sciences University of Maryland School of Medicine Baltimore Maryland USA; ^2^ Department of Epidemiology and Public Health University of Maryland School of Medicine Baltimore Maryland USA; ^3^ Department of Medicine Division of Gerontology Geriatrics and Palliative Medicine, Baltimore VAMC University of Maryland School of Medicine Geriatric Research Education and Clinical Center (GRECC) VA Maryland Health Care System Baltimore Maryland USA; ^4^ Intramural Research Program National Institute on Aging National Institutes of Health Baltimore Maryland USA

**Keywords:** cancer, censoring, competing risks, sensitivity analysis, time‐to‐event analysis

## Abstract

**INTRODUCTION:**

Cancer is inversely associated with cognitive impairment. Whether this is due to statistical handling of attrition (death and censoring) is unknown.

**METHODS:**

We quantified associations between cancer history and incident cognitive impairment among Health, Aging, and Body Composition Study participants without baseline cognitive impairment or stroke (*n *= 2604) using multiple competing‐risks models and their corresponding estimands: cause‐specific, subdistribution, and marginal hazards, plus composite‐outcome (cognitive impairment or all‐cause mortality) hazards. All‐cause mortality was also modeled.

**RESULTS:**

After covariate adjustment (demographics, apolipoprotein E ε4, lifestyle, health conditions), cause‐specific and marginal hazard ratios (HRs) were similar to each other (≈ 0.84; *P* values < 0.05). The subdistribution HR was 0.764 (95% confidence interval [CI] = 0.645–0.906), and composite‐outcome Cox model HR was 1.149 (95% CI = 1.016–1.299). Cancer history was positively associated with all‐cause mortality (HR = 1.813; 95% CI = 1.525–2.156).

**DISCUSSION:**

Cause‐specific, subdistribution, and marginal hazards models produced inverse associations between cancer and cognitive impairment. Competing risk models answer slightly different questions, and estimand choice influenced findings here.

**Highlights:**

- Cancer history is inversely associated with incident cognitive impairment.
- Findings were robust to handling of competing risks of death.
- All models also addressed possible informative censoring bias.
- Cancer history was associated with 16% lower hazard of cognitive impairment.
- Cancer history was associated with 81% higher all‐cause mortality hazard.

## 1 BACKGROUND

Many epidemiologic studies have reported that cancer history is inversely associated with risk of cognitive impairment, dementia, or Alzheimer's disease (AD).^1^, ^2^, ^3^, ^4^, ^5^, ^6^, ^7^, ^8^, ^9^, ^10^, ^11^, ^12^, ^13^, ^14^, ^15^, ^16^, ^17^, ^18^, ^19^, ^20^, ^21^, ^22^, ^23^ This finding has been repeatedly,^1^, ^2^, ^3^, ^4^, ^5^, ^6^, ^7^, ^8^, ^9^, ^10^, ^11^, ^12^, ^13^, ^14^, ^15^, ^16^, ^17^, ^18^, ^19^, ^20^, ^21^, ^22^, ^23^ though not universally,^3^, ^24^, ^25^, ^26^ replicated across multiple populations, but nevertheless remains counterintuitive because cancer treatment negatively affects cognition.^27^ Biological mechanisms for an inverse relationship between cancer and cognitive impairment are unknown and likely multi‐faceted. Researchers posit that cancer and cognitive impairment are consequences of opposite extremes of a common etiology, where dysregulation in one direction promotes uncontrolled cell growth and higher cancer risk, and dysregulation in the opposite direction promotes neuronal cell death that contributes to cognitive impairment and dementia.^28^, ^29^, ^30^, ^31^

An alternative explanation is that these inverse associations are due to attrition biases.^15^ For example, persons diagnosed with cancer are more likely to die or drop out of a study before onset or diagnosis of cognitive impairment or dementia than persons without cancer. Death is a competing risk of cognitive impairment, for which survivors may differ by factors that induce confounding between cancer and cognitive impairment (i.e., selective survival bias).^32^ Survivors lost to follow‐up and who have censored cognitive impairment status may be sicker than those who remain (i.e., informative censoring bias).^32^ These attrition biases can distort estimates toward stronger negative associations, even if cancer and cognitive impairment are truly unrelated or positively associated.^32^

Multiple time‐to‐event models with different estimands (targets of estimation) that answer slightly different questions can address competing risks of death. The most common estimand when studying cancer and cognitive impairment, dementia, or AD^1^, ^2^, ^3^, ^4^, ^6^, ^8^, ^9^, ^12^, ^13^, ^19^, ^23^, ^24^, ^25^, ^26^ is the cause‐specific hazard ratio (HR), which has been suggested for etiologic research among survivors, and is often estimated using Cox models.^33^, ^34^, ^35^, ^36^ A less common estimand in this context is the subdistribution hazard, often estimated using the Fine–Gray model, which can be transformed into cumulative incidences.^33^, ^34^, ^35^, ^36^, ^37^ Instead of conditioning on survival, this estimand addresses competing risks by treating participants who die before cognitive impairment onset as deterministically free of cognitive impairment after death and retains them for follow‐up. That is, death is not “eliminated” as a competing risk, but a reason why some participants do not develop cognitive impairment; therefore, this approach has been suggested for prognostic research.^33^, ^36^ One study on cancer and AD using administrative claims data to compare cause‐specific and subdistribution HRs found subdistribution HRs to have larger inverse associations due to higher mortality rates in participants with cancer.^12^

Unlike cause‐specific and subdistribution HRs, marginal HRs^33^ treat death as a censoring event to be eliminated. This estimand is interpreted as the association between cancer and cognitive impairment eliminating death as a competing event and has been suggested for etiologic research not restricted to survivors.^33^ A final estimand is the composite‐outcome HR, for which the event of interest is incident cognitive impairment or all‐cause mortality. Marginal and composite‐outcome HRs are uncommon in studies of cancer and cognitive impairment; however, composite outcomes are common in randomized trials to preserve the baseline cohort and assess overall harm^38^ (see Methods S1, Table S1 in supporting information).

**RESEARCH‐IN‐CONTEXT**

1. **Systematic review**: Studies have consistently found cancer to be inversely associated with cognitive impairment and dementia. Little work has formally compared statistical models to address attrition biases (informative censoring and selective survival from competing risks of death). Using data from the Health, Aging, and Body Composition Study, we addressed informative censoring via weighted analysis and compared competing‐risks models and their estimands.
2. **Interpretation**: Cause‐specific hazard ratios (HRs), which condition on survival, and marginal HRs, which treat death as censoring, were similar; cancer history was associated with 16% lower cognitive impairment hazard. Subdistribution HRs, which are not easily interpretable, suggested stronger inverse associations, because deaths without cognitive impairment were treated as “at risk,” and cancer history was positively associated with all‐cause mortality.
3. **Future directions**: Findings highlight the importance of model/estimand choice when investigating associations between cancer and cognitive impairment. Rigorous handling of attrition biases is needed to investigate biological mechanisms.

Regardless of the chosen estimand, which follows from the research question,^39^ losses to follow‐up among survivors can bias estimates of the association between cancer and incident cognitive impairment. Inverse‐probability weights (IPWs) for censoring can mitigate potential informative censoring bias.^32^ Although IPWs for censoring can be implemented when estimating any of the HRs described, they have not been used when comparing HRs to study cancer and cognitive impairment.

This study aims to compare estimated associations of cancer history with incident cognitive impairment between competing‐risks models and their corresponding estimands (cause‐specific, subdistribution, marginal, and composite‐outcome hazards) while also addressing informative censoring via IPWs for censoring. Although simulation studies have demonstrated that the competing risk of death and selective survival bias cannot fully explain inverse associations between cancer and dementia,^40^ we hypothesize that findings will differ by model and corresponding estimand, and estimates weighted to address attrition biases will be closer to the null than estimates from published meta‐analyses (0.59—0.89).^5^, ^7^, ^15^, ^22^

## 2 METHODS

### 2.1 Participants

Participants included older Black and White men and women enrolled in the Health, Aging, and Body Composition (Health ABC) Study. The design and conduct of Health ABC are described elsewhere.^41^, ^42^ Briefly, 3075 participants (41.7% Black; 51.5% women) aged 70 to 79 years were recruited between April 1997 and June 1998 by mailing to a random sample of White and all Black Medicare‐eligible adults living in selected zip codes around two metropolitan areas (Pittsburgh, Pennsylvania and Memphis, Tennessee). Eligibility criteria included reporting no difficulty walking a quarter mile, climbing 10 stairs, or performing basic activities of daily living; no life‐threatening illness; and no plans to leave the area for 3 years. All participants provided written informed consent. The study was approved by institutional review boards at both participating sites. Enrolled participants were assessed at annual follow‐up visits. The enrollment visit (“year 1”) was deemed baseline. The present study focused on cancer history, incident cognitive impairment, and all‐cause mortality through the tenth study year (2007 to 2008). Participants were excluded if they had cognitive impairment (defined in the next section) or reported a history of stroke at enrollment. The final analytic sample included 2604 participants (37.9% Black; 52.3% women).

### 2.2 Outcomes: cognitive impairment and all‐cause mortality

Outcomes for the present analysis were determined through the 10th annual visit (9 years after baseline). Analogous to previous studies of Health ABC participants,^43^ cognitive impairment at baseline was defined as having a Teng Modified Mini‐Mental Score (3MS) < 78 or a presence of the medications galantamine, rivastigmine, memantine, donepezil, or tacrine in the medication inventory assessment.^44^, ^45^ Incident cognitive impairment at follow‐up was defined as 3MS < 78, presence of medications, or decline in 3MS > 1.5 standard deviations of baseline 3MS.^44^ Assessments of 3MS occurred at years 1, 3, 5, 7, 8, 9, and 10. Medication inventory was assessed at years 1,2, 3, 5, 6, 8, and 10. Of the 739 incident cognitive impairment events, 335 were based on 3MS < 78, 348 were based on 3MS decline, and an additional 56 were based on medications. All‐cause mortality was determined using semiannual surveillance and obituary review. A committee determined date of death using hospital records, death certificates, informant interviews, and autopsy reports. Days of follow‐up were calculated as number of days from enrollment to date of death, last day of contact, or the actual or projected date of the 10‐year visit. There were 661 deaths by year 10. These data were used to determine vital status at each scheduled annual visit to identify whether missing cognitive impairment status was due to death or loss to follow‐up.

### 2.3 Exposure: cancer history

Cancer history at each visit was operationally defined as cancer of any type except non‐melanoma skin cancer at the current or past visit. As previously described,^46^ prevalent cancer history status at enrollment was based on self‐report or administrative claims, and incident cancer history at follow‐up was based on Health ABC cancer event data. Specifically, participants or their proxies were asked to report any new cancer‐related hospitalizations or outpatient events; and approximately every 6 months they were asked direct questions to elicit information about these types of events since the last contact. An incident cancer‐related event during follow‐up prompted an adjudication protocol that included dated hospital records such as pathology and cytology reports and radiologic and laboratory data. Days until incident cancer diagnosis during follow‐up were calculated as number of days from enrollment to first cancer event. These data were used to determine cancer history status at each scheduled annual visit. Participants who had cancer history at enrollment were considered prevalent cancer history cases who were positive for cancer history at all visits. Participants who did not have prevalent cancer history at enrollment, but were diagnosed with cancer during follow‐up, were considered incident cancer history cases who were negative for cancer history until their first visit with a cancer diagnosis and positive from diagnosis through follow‐up. Cancer history (lagged one visit [year] to ensure temporal ordering) was modeled as a time‐varying exposure in statistical analyses (described in section 2.5.2).

### 2.4 Covariates

Covariates included age (years), sex, self‐reported race (Black or White), education (high‐school graduate or not), marital status (currently married; never married; or separated, widowed, or divorced), study site (Memphis or Pittsburgh), apolipoprotein E (*APOE*) ε4 carrier, self‐reported physical activity (characterized as sedentary, moderately active, or highly active), renal disease determined using estimated glomerular filtration rate (eGFR) measured using cystatin C at the enrollment visit (eGFR_CysC _< 60 mL/min/1.73 m^2^, where eGFR_CysC_ = 76.7 × cystatin C^−1.19^),^47^ smoking status (never, former, current), alcohol intake (drinks/week), and depressive symptoms measured using the Centers for Epidemiological Studies Depression (CES‐D) scale.^48^ These covariates were assessed at enrollment and treated as time ‐invariant in all statistical analyses.

Covariates also included health conditions assessed at enrollment and follow‐up visits that were treated as time varying in primary statistical analysis (see section 2.5.2). Diabetes history was defined as fasting glucose ≥ 126 mg/dL, self‐report, or antidiabetes medication use. Hypertension history was defined as self‐report or use of medication (beta blockers, alpha blockers, ace inhibitors, hypotensive agents, angiotensin II, hydralazine, calcium channel blockers; and loop, thiazide, and other miscellaneous diuretics). Cardiovascular disease history at enrollment was defined as presence of coronary heart disease (self‐report, administrative claims for heart bypass, myocardial infarction, or medication for angina) or cerebrovascular disease (self‐reported transient ischemic attack). Days until incident cardiovascular disease during follow‐up were calculated as number of days from enrollment to diagnosis. Incident cardiovascular disease was defined as hospitalization for myocardial infarction, angina, or stroke and was adjudicated. These data were used to determine cardiovascular disease status at each scheduled annual visit. Time‐varying diabetes history, hypertension history, and cardiovascular disease history were operationalized similar to cancer history (see section 2.3), and these covariates were lagged one visit (year) in statistical analysis (see section 2.5.2).

### 2.5 Statistical analysis

#### 2.5.1 Descriptive analysis

Comparisons between participants with and without prevalent (baseline) cancer history were made using *t* tests and chi‐square or Fisher exact tests for baseline continuous and categorical covariates, respectively. Cumulative incidence of all‐cause mortality was computed using one minus the Kaplan–Meier survival curve estimate and compared between participants with and without prevalent cancer history using the log‐rank test. Cumulative incidences of cognitive impairment for competing risks were computed for participants with and without prevalent cancer history and compared using the Gray test.^49^ The cumulative incidence of cancer history, which accounted for prevalent cases at baseline, was similarly computed.

#### 2.5.2 Primary analysis: time‐to‐event models

We examined the association of time‐varying cancer history with incident cognitive impairment using four time‐to‐event models:^33^, ^37^ cause‐specific hazards regression, subdistribution hazards regression, marginal hazards regression, and composite outcome (cognitive impairment or all‐cause mortality) hazards regression.^33^, ^34^, ^35^, ^37^ The corresponding estimands of these models treat death as, respectively, a conditioning term (removed from risk set), a non‐event (retained in the risk set, but not eliminated as a competing risk), a form of censoring (eliminated as a competing risk), or part of the outcome.^33^, ^34^, ^35^, ^36^, ^37^ (See Methods S1 and Table S1 for additional detail and formal expressions of hazard estimands and corresponding HRs.) We used modified pooled Poisson regression with robust standard errors in all models^50^ to address discrete time incident cognitive impairment.^51^ Owing to cognition variables not being assessed at all visits, some cases of incident cognitive impairment were interval censored. We addressed this issue using the logarithm of time (years since last visit) as an offset term.

**FIGURE 1:**
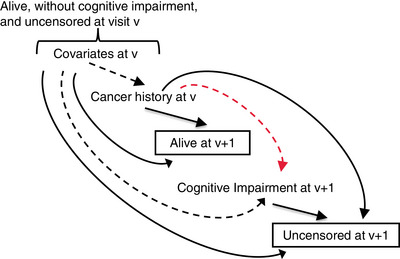
Directed acyclic graph conveying selective survival and informative censoring biases. Dashed lines are relationships distorted by conditioning on being alive and uncensored. Red line is the relationship of interest. Variable *v* refers to visit number.

Let *v* denote visit (study year) number, where *v* = 2,…,10. For all analyses, we considered the following temporal order of realization of relevant time‐varying variables: covariates at *v –* 1, cancer history status at *v –* 1, vital status at *v*, cognitive impairment at *v*, censoring at *v*, covariates at *v*, and so on. All models involved regressing incident cognitive impairment at *v* on cancer history status and time‐varying covariates at *v –* 1 and baseline covariates. We also used this temporal ordering to inform strategies to address missing covariates, death, and censoring. These sources of attrition were addressed using IPWs, but implementation differed depending on the estimand. The directed acyclic graph in Figure 1 conveys potential selective survival and informative censoring biases that can occur by restricting analysis to those who are alive and uncensored, respectively. Depending on the research question,^39^ conditioning on survival can distort the association between covariates and cancer history, inducing unmeasured confounding if these covariates are not included in analysis. Conditioning on being uncensored can also distort the association between cancer history and cognitive impairment and induce unmeasured confounding. IPWs aim to overcome these distortions.

For all time‐to‐event models, IPWs to address missing baseline covariates were constructed from probabilities estimated by logistically regressing an indicator for complete baseline covariates on select baseline covariates and interaction terms (see Methods S2 in supporting information). IPWs to address censored cognition status were constructed from probabilities estimated by logistically regressing an indicator for observed (non‐censored) cognition status on cancer history (prevalent and incident cancer history cases separated), other time‐varying health conditions (hypertension history, diabetes history, and cardiovascular disease history), visit number, baseline covariates, and select interaction terms (see Methods S2) among those with complete baseline covariates who were still in the risk set, which depended on the time‐to‐event model and its corresponding estimand (see Methods S1, Table S1). All IPWs were stabilized using probabilities from regressions of relevant indicators on age, sex, race, and study site among those in the model‐specific risk set; and all stabilized IPWs were truncated at 0.5% and 99.5% to address large weights.^52^ For all analyses, statistical significance was defined as a *P* value < 0.05 or a 95% confidence interval that excludes the null. R Statistical Software version 3.6.1 was used for all analyses.

In primary analysis, covariates in all hazard regression models included study site, sex, age, race, education, marital status, *APOE* ε4 carrier, smoking, alcohol intake, diabetes history, hypertension history, cardiovascular disease history, renal disease, CES‐D score, and baseline 3MS score. We additionally included interactions of race with diabetes history, hypertension history, cardiovascular disease history, renal disease, *APOE* ε4 carrier, and 3MS; *APOE* ε4 carrier with smoking status; study site with 3MS; and all two‐ and three‐way interactions between race, age, and education. Interactions were selected based on previously studied subgroups with potentially different normative 3MS performance.^53^ Moreover, including interactions increases covariate modeling flexibility that may also provide additional protection against attrition biases, as described by Kalbfleisch and Prentice^54^ (chapter 8.2.5).

Cause‐specific hazards regression estimates associations conditioned survival. At each visit *v*, the final IPW used in this model was a product of the IPW for baseline covariates and cumulative product of IPW for censoring up to visit *v* among participants who had complete covariates, who were uncensored and without cognitive impairment at visit *v –* 1, and who were alive at visit *v*.^33^

Subdistribution hazards regression treats participants who died without cognitive impairment as deterministically free of cognitive impairment, but retains these participants in the risk set and does not eliminate the competing risk.^33^, ^37^ The rationale of this approach is to enable transformation of hazards to cumulative incidence; but this advantage is only available with time‐invariant exposures and covariates, thus it provides no such benefit in the present analysis.^55^, ^56^ At each visit *v*, the final IPW used in this model was a product of the IPW for baseline covariates and the cumulative product of IPW for censoring up to visit *v* among those with complete covariates who were uncensored and without cognitive impairment at *v –* 1. Once a participant died, the contribution to the IPW cumulative product for censoring equaled 1, because participants cannot be censored after they die.^33^, ^37^

Marginal hazards regression estimates associations that treat the competing risk of death as a form of censoring.^33^ Thus, the HR estimands are interpreted as associations as if the competing risk was eliminated by creating, through weighting, a pseudopopulation in which death is independent of factors that may affect cognitive impairment risk. That is, weighting creates a hypothetical population in which no one died over follow‐up. Because death, like other forms of censoring, is likely informative, IPWs for death were constructed from probabilities estimated by logistically regressing an indicator for being alive on the same covariates and select interaction terms that were included in censoring IPW model (see section 2.5.2 and Methods S2) among those with complete baseline covariates who were still in the risk set. The final IPW at each visit *v* was a product of the IPW for baseline covariates; cumulative product of IPW for death up to visit *v* among those with complete covariates who were alive, uncensored, and without cognitive impairment at *v –* 1; and cumulative product of IPW for censoring up to visit *v* among those with complete covariates, who were uncensored and without cognitive impairment at *v –* 1 and who were alive at *v*.^33^

Composite‐outcome hazards regression changes the event from incident cognitive impairment to incident cognitive impairment or death (all‐cause mortality). The final IPW at each visit *v* was a product of the IPW for baseline covariates and the cumulative product of IPW for censoring up to visit v among those with complete covariates who were uncensored and without cognitive impairment and alive at *v –* 1.

#### 2.5.3 Additional analysis and sensitivity analyses

To facilitate interpretation of results using the subdistribution hazards or composite outcome, we additionally performed analysis with all‐cause mortality as the outcome, as is recommended for studies with competing risks.^57^ The only IPW for this model was that for baseline covariates. In addition to the primary analysis described in section 2.5.2, we performed multiple sensitivity analyses. First, we refit the primary analysis models without IPWs for censoring and missing covariates to determine robustness of findings to assumptions about censoring and missingness. Note that IPWs for death were still used to estimate marginal HRs, because these weights are crucial for differentiating marginal from cause‐specific HRs (see Methods S1).^33^ Next, we performed a sensitivity analysis to address two other potential sources of bias, baseline selection bias and overadjustment bias.^15^ In this analysis, we restricted the sample to participants who did not have prevalent cancer history at enrollment. Enrolled participants who survived cancer were likely healthier and not representative of the overall population who had a history of cancer during recruitment, thereby creating a selection bias.^15^ Also, we only included baseline covariates in hazard regression models and did not treat diabetes, hypertension, or cardiovascular disease history as time varying. Cancer is known to be associated with incidence of these conditions;^15^ therefore, they may mediate rather than confound the association between cancer history and incident cognitive impairment, which could lead to an overadjustment bias. Therefore, sensitivity analysis of each hazard regression model was performed three ways: (1) restricted to participants who did not have prevalent (baseline) cancer history only, (2) restricted to analysis with baseline covariates only, and (3) restricted both to participants who did not have prevalent cancer history *and* to analysis with baseline covariates. A final sensitivity analysis addressed the 3MS assessment schedule. Because 3MS was assessed at years 7 and 9 only among participants who were included in the Health ABC Cognitive Vitality Substudy,^58^ we repeated primary analysis by ignoring 3MS assessments carried out in years 7 and 9 to address this potential selection bias.

## 3 RESULTS

**FIGURE 2:**
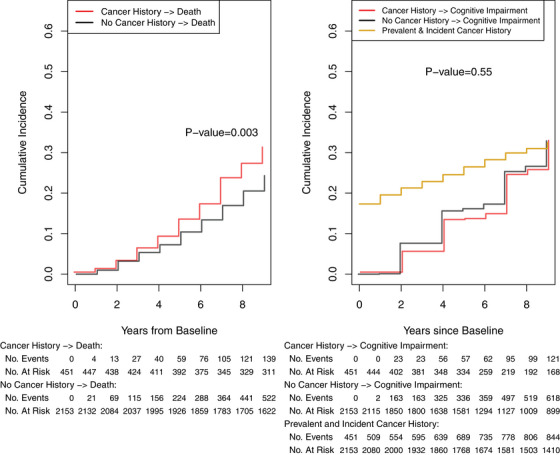
Nine‐year cumulative incidence of all‐cause mortality (death), cognitive impairment, and combined prevalence and cumulative incidence of cancer history. Left: Cumulative incidence of all‐cause mortality by prevalent (baseline) cancer history status. *P* value from logrank test. Table shows cumulative number of deaths and number remaining at risk. Right: Cumulative incidence of cognitive impairment by prevalent cancer history status. *P* value from a Gray test. Table shows cumulative number of incident cognitive impairment cases and number remaining at risk. Combined prevalence and cumulative incidence of cancer history. Table shows cumulative number of prevalent and incident cancer history cases and number remaining at risk.

Among 2604 participants, 451 (17.3%) had prevalent cancer history at enrollment (baseline). Those with prevalent cancer history were older, less likely to be female or Black, and more likely to have renal disease than participants who did not have prevalent cancer history (Table 1). Participants with prevalent cancer history had a higher 9‐year cumulative incidence of all‐cause mortality than participants without prevalent cancer history (30.9%; 95% confidence interval [CI] 26.5–35.0% vs. 24.3%; 95% CI 22.5–26.1%; *P* value = 0.003; Figure 2, Table S2 in supporting information). Participants with and without prevalent cancer history had similar 9‐year cumulative incidences of cognitive impairment (32.5%, 95% CI 27.6–37.4% vs. 32.9%, 95% CI 30.7–35.1%; *P* value = 0.55; Figure 2, Table S3 in supporting information). There were 844 cancer history cases over the 9‐year follow‐up, and the combined prevalence and cumulative incidence of cancer history was 32.5% (95% CI 30.7–34.3%; Figure 2, Table S4 in supporting information).

**TABLE 1:** Baseline characteristics of 2604 participants enrolled in the Health, Aging, and Body Composition Study without cognitive impairment at baseline.

	Prevalent cancer History (*N* = 451)	No prevalent cancer History (*N* = 2153)	
Characteristic	Mean (SD) or n/N (%)	Mean (SD) or n/N (%)	*p* value^*^
Age (years)	74.0 (2.9)	73.5 (2.8)	0.001
Female	200/451 (44.3)	1163/2153 (54.0)	< 0.001
Black race	143/451 (31.7)	845/2153 (39.2)	0.003
*APOE* ε4 carrier	112/451 (24.8)	561/2153 (26.1)	0.63
High school graduate	373/451 (82.7)	1704/2149 (79.3)	0.11

	*N *= 451	*N* = 2153	
Physical activity:			0.057
Highly active	35 (7.8)	145 (6.7)	
Moderately active	245 (54.3)	1060 (49.2)	
Sedentary	171 (37.9)	948 (44.0)	
Alcohol intake (drinks/week)	2.15 (1.48)	2.14 (1.57)	0.93

	*N* = 451	*N* = 2152	
Smoking status:			0.056
Current smoker	34 (7.5)	206 (9.6)	
Former smoker	230 (51.0)	970 (45.1)	
Never smoker	187 (41.5)	976 (45.4)	

	*N *= 438	*N *= 2077	
Marital status:			0.21
Married	263 (60.0)	1155 (55.6)	
Widowed/divorced/separated	153 (34.9)	819 (39.4)	
Never married	22 (5.0)	103 (5.0)	
Centers for Epidemiological Studies—Depression score	4.75 (4.92)	4.54 (5.26)	0.44
Diabetes	86/451 (19.1)	373/2153 (17.3)	0.41
Renal disease	219/451 (48.6)	907/2153 (42.1)	0.014
Hypertension	196/451 (43.5)	905/2153 (42.0)	0.61
Teng Modified Mini‐Mental Score	92.2 (5.4)	91.8 (5.6)	0.18

	*N *= 451	*N* = 2153	
Cardiovascular disease:			0.30
Coronary heart disease	95 (21.1)	412 (19.1)	
Other cardiovascular disease	7 (1.6)	20 (0.9)	
No cardiovascular disease	349 (77.4)	1721 (79.9)	

	*N* = 451	*N* = 2153	
Study site:			0.92
Memphis	220 (48.8)	1059 (49.2)	
Pittsburgh	231 (51.2)	1094 (50.8)	

Abbreviations: *APOE*, apolipoprotein E; SD, standard deviation.

* *P* values from t tests, chi‐square tests, or Fisher exact tests.

In primary analysis, the cause‐specific, marginal, and subdistribution hazards of incident cognitive impairment were all significantly lower for participants with cancer history than for participants without cancer history (*P* value < 0.05) after adjustment for covariates (Table 2). Of these three estimates, the subdistribution HR had the largest magnitude, with cancer history associated with 23.6% lower hazard of cognitive impairment (HR = 0.764, 95% CI 0.645–0.906; *P* value = 0.002). The cause‐specific HR had the smallest magnitude, which was 15.6% lower for participants with versus without cancer history (HR = 0.844, 95% CI 0.718–0.992; *P* value = 0.039) and similar to the marginal HR (HR = 0.841, 95% CI 0.715–0.989; *P* value = 0.037). In contrast, cancer history was significantly associated with 14.9% higher hazard of the composite outcome (HR = 1.149, 95% CI 1.016–1.299; *P* value = 0.026) and significantly associated with 81.3% higher hazard of all‐cause mortality (HR = 1.813, 95% CI 1.525–2.156; *P* value < 0.001). Results were similar to those without IPWs for missing covariates and censoring (Table S5 in supporting information).

**TABLE 2:** Association of time‐varying cancer history with incident cognitive impairment and all‐cause mortality.

		Primary analysis:				Sensitivity analysis:			
		All cancer cases, time‐varying covariates^a^, ^c^		Incident cancer cases only, time‐varying covariates^b^, ^c^		All cancer cases, baseline covariates only^a^, ^d^		Incident cancer cases only, baseline covariates only^b^, ^d^	
Outcome	Discrete‐time model	HR (95% CI)	*p* value	HR (95% CI)	*p* value	HR (95% CI)	*p* value	HR (95% CI)	*p* value
Cognitive impairment	Cause‐specific hazard	0.844 (0.718, 0.992)	0.039	0.648 (0.441, 0.951)	0.027	0.848 (0.723, 0.994)	0.042	0.647 (0.443, 0.944)	0.024
	Marginal hazard	0.841 (0.715, 0.989)	0.037	0.650 (0.440, 0.960)	0.030	0.846 (0.720, 0.993)	0.040	0.649 (0.442, 0.953)	0.027
	Subdistribution hazard	0.764 (0.645, 0.906)	0.002	0.462 (0.308, 0.692)	< 0.001	0.767 (0.648, 0.908)	0.002	0.463 (0.310, 0.691)	< 0.001
Composite^e^	Cox hazard	1.149 (1.016, 1.299)	0.026	1.385 (1.105, 1.737)	0.005	1.146 (1.015, 1.295)	0.027	1.369 (1.092, 1.716)	0.006
All‐cause mortality	Cox hazard	1.813 (1.525, 2.156)	< 0.001	3.160 (2.468, 4.044)	< 0.001	1.804 (1.517, 2.145)	< 0.001	3.103 (2.423, 3.974)	< 0.001

Abbreviations: CI, confidence interval; HR, hazard ratio.

^a^ Includes *N* = 2604 participants.

^b^ Includes *N* = 2153 participants (excludes 451 participants who had prevalent (baseline) cancer history).

^c^ Adjustment for time‐varying diabetes history, hypertension history, and cardiovascular disease history.

^d^ Adjustment for prevalent (baseline) diabetes history, hypertension history, and cardiovascular disease history.

^e^ Composite outcome of incident cognitive impairment or all‐cause mortality.

In a sensitivity analysis in which 451 participants with prevalent cancer history were removed and adjustment was for baseline (rather than time‐varying) diabetes history, hypertension history, and cardiovascular disease history, the magnitudes of all estimated associations increased. Notably, the subdistribution HR for cognitive impairment comparing those with to those without cancer history was 0.463 (95% CI 0.310–0.691; *P* value < 0.001) and the HR for all‐cause mortality was 3.103 (95% CI 2.423–3.974; *P* value < 0.001; Table 2). These findings were similar to those obtained after removing participants with prevalent cancer history while adjusting for time‐varying covariates. However, when participants with prevalent cancer history were retained, but adjustment was only for baseline covariates, findings were similar to those from the primary analysis (Table 2). Estimates from analysis that ignored 3MS assessments at years 7 and 9 were similar to those from the primary analysis (Table S6 in supporting information).

## 4 DISCUSSION

Overall, findings from primary and sensitivity analyses of cause‐specific, subdistribution, and marginal hazards regression demonstrated an inverse association between cancer history and incident cognitive impairment. Estimated cause‐specific and marginal HRs were similar, suggesting that the inverse association was not explained by the influence of measured covariates on mortality. Moreover, estimates from these two models were within the range of previous estimates derived from large meta‐analyses (0.59–0.89),^5^, ^7^, ^15^, ^22^ even after addressing potential selection bias from prevalent cancer history cases and enrollment in a substudy, and after addressing potential overadjustment bias from time‐varying disease covariates.^15^ Indeed, magnitudes of association were stronger after removing prevalent cancer history cases, which is consistent with the negligible difference in cumulative incidence of cognitive impairment between participants with and without prevalent cancer history. One reason for this finding may be that enrolled baseline cancer history cases are likely healthier than the general population of cancer cases (i.e., more likely to have been successfully treated) and thus may have a physiologic profile more like cancer‐free participants than participants with incident cancer; therefore, their inclusion may have diluted the association between cancer history and cognitive impairment. Models of other estimands proposed to handle competing risks, such subdistribution HRs and HRs of composite outcomes, produced estimates that were respectively stronger than and opposite from those from cause‐specific and marginal HR estimation, owing to different interpretations due to their different handling of the competing risk of death, which can be seen in the notation provided in Methods S1 and Table S1. Both findings can be explained by the positive association between cancer history and all‐cause mortality.^33^

Subdistribution HRs, which do not eliminate the competing risk of death, are generally difficult to interpret, especially for etiologic research, because deaths without cognitive impairment are retained in the risk set even though they cannot subsequently develop cognitive impairment. However, subdistribution HRs are nonetheless commonly used to transform HRs into estimated cumulative incidence curves when examining time‐invariant (e.g., prevalent baseline) exposures. Subdistribution hazard models are not commonly applied to studies of cancer and cognitive impairment because investigators are often interested in modeling incident cancer history cases over time, not just (or instead of) prevalent cases at baseline. In this instance, subdistribution hazard ratios cannot easily be transformed into cumulative incidence curves.^55^, ^56^

The current study innovatively adds to the body of evidence on the inverse association between cancer and cognitive impairment by (1) comparing estimands that vary in their interpretation and answer different research questions due to how they handle competing risks,^39^ and (2) addressing two types of attrition bias, selective survival bias and informative censoring bias. We also addressed selection bias due to incomplete baseline covariates, and we demonstrated that findings were robust to potential selection bias and overadjustment bias.^15^ This study also empirically demonstrated the limitations of subdistribution HRs and composite outcomes as estimands for addressing competing risks when associations of an exposure (cancer history) with an outcome (incident cognitive impairment) and all‐cause mortality are in opposite directions, particularly when scientific interest is in understanding biological mechanisms (etiology).^12^, ^33^

Multiple biological mechanisms have been proposed to explain inverse associations of cancer with cognitive impairment, dementia, and AD. Evidence related to energy metabolism suggests that higher oxidative phosphorylation and lower glycolysis relate to cognitive decline and dementia,^59^, ^60^, ^61^, ^62^, ^63^ whereas glycolysis is upregulated in cancer.^64^ An extensive literature review^29^ delineated multiple biological factors that are downregulated in AD, but upregulated in cancer, such as expression of estrogen, neurotrophins and growth factors, the *PI3K/AKT/MTOR* pathway, vimentin, carbonic anhydrases, and ubiquitin. Recent work has identified *PIN1* polymorphisms to be of interest for suppressing formation of tau tangles, but contributing to cell proliferation in cancer.^65^, ^66^ Conversely, apoptosis pathways such as the P53 protein are upregulated in AD and downregulated in cancer.^29^

This study has multiple strengths. First, study participants were part of a large, well‐characterized cohort that included measurement of multiple relevant variables and excluded participants with baseline cognitive impairment or history of stroke. Second, we rigorously addressed multiple sources of selection bias using IPWs including missing covariates and censoring. Third, we compared multiple estimands proposed to address competing risks, including marginal hazards, which treated mortality as a form of censoring addressed using IPWs. Fourth, we implemented sensitivity analyses that were informed by the literature^15^ to demonstrate robustness of findings to multiple potential sources of bias. Last, a potential concern is that cancer history status may influence differential delay of dementia or AD diagnosis, leading to diagnostic bias.^15^, ^67^ The present study overcomes this concern by relying primarily on measures of global cognition assessed at regular pre‐scheduled visits. Moreover, a recent study using electronic health record data for dementia diagnosis indicates that diagnostic bias is toward positive rather than negative associations (e.g., persons with cancer likely receive earlier dementia diagnosis owing to more frequent health‐care use).^24^

Despite the above strengths, this study had important limitations to consider. First, Health ABC is an observational cohort study comprising Black and White older adults in two US metropolitan areas; therefore, findings cannot be generalized to other races or geographical regions, and there is potential for unmeasured confounders and contributors of attrition bias. Indeed, unmeasured confounders may include biological mechanisms that contribute to the inverse association between cancer history and cognitive impairment. Second, associations between cancer history and cognitive impairment were quantified using HRs, which do not generally have a causal interpretation.^32^, ^68^ Third, mild cognitive impairment and dementia were not differentiated or adjudicated using a comprehensive neuropsychological battery; however, we would not expect a misclassification bias from 3MS to differ by cancer history status; thus, misclassification would plausibly bias findings toward the null. Fourth, we did not distinguish cancer types; however, we did remove non‐melanoma skin cancer, which has lower mortality risk than other commonly studied cancers (e.g., breast, prostate, or colorectal cancer), yet had a stronger inverse association with dementia than these cancers in a recent meta‐analysis.^15^ Last, the use of medications may have overestimated cognitive impairment cases due to multiple medication indications. Medication‐only cases in the present study comprised only 7.6% of incident cognitive impairment cases, and thus could not contribute to sufficient diagnostic bias (which would more plausibly be toward positive associations^24^) to explain the inverse associations. Moreover, 3MS and medications have been successfully used to assess cognitive impairment and dementia in previous Health ABC research.^43^

In summary, we found robust evidence for an inverse association between cancer history and incident cognitive impairment. We recommend that investigators who are interested in answering etiologic questions about the association between cancer history and incident cognitive impairment estimate both cause‐specific hazards and marginal hazards as strategies to address competing risks and investigate selective survival bias. Future research includes replication in studies with adjudicated dementia diagnosis and simultaneous empirical investigation of biological mechanisms that are hypothesized to contribute to inverse associations using modern causal inference methods.^33^

## CONFLICT OF INTEREST STATEMENT

The authors have no conflicts of interest to declare. Author disclosures are available in the supporting information.

## CONSENT STATEMENT

The present study was approved by the institutional review board of the University of Maryland Baltimore (^#^HP‐00103166). Research was conducted in accordance with the Declaration of Helsinki and its later amendments. All participants provided written informed consent.

## DIVERSITY, EQUITY, AND INCLUSION STATEMENT

The Health ABC Study was established in the late 1990s and was innovative at the time for recruiting approximately even numbers of Black and White men and women aged 70 to 79 years. Other races and ethnicities were not recruited; we have acknowledged and discussed this limitation of our study.

## Supporting information



Supporting Information

Supporting Information
