# Supplementary material for: The inverse association between cancer history and incident cognitive impairment: Addressing attrition bias
Source: Alzheimers Dement. 2024 Sep 26;20(11):7902–12. doi: 10.1002/alz.14268 (PMC11567823; doi:10.1002/alz.14268)
Supplement: Supplementary file 1 — Supporting Information [file ALZ-20-7902-s001.docx]

**Supplementary Material**

The inverse association between cancer history and incident cognitive impairment: addressing attrition bias

**Methods**

1. **Formulas for cause-specific, marginal, subdistribution, and composite-outcome hazards**
2. **Inverse probability weight (IPW) models**

**Supplementary Tables**

**Supplementary Table 1.** Hazard ratio (HR) estimands, notation, and their interpretation.

**Supplementary Table 2.** Cumulative incidence of all-cause mortality by prevalent (baseline) cancer history status.

**Supplementary Table 3.** Cumulative incidence of cognitive impairment by prevalent (baseline) cancer history status.

**Supplementary Table 4.** Prevalence and cumulative incidence of cancer history.

**Supplementary Table 5.** Association of cancer history with incident cognitive impairment and all-cause mortality without inverse-probability weights for censoring and missing covariates.

**Supplementary Table 6.** Association of cancer history with incident cognitive impairment and all-cause mortality without Teng Modified Mini-Mental State^1^ assessments at years 7 and 9.

**Methods**

1. **Formulas for cause-specific, marginal, subdistribution, and composite-outcome hazards**

To express the formulas for the hazard estimands assessed in this study, we first define some notation. Let v = 1, 2,…,V denote visit numbers, where v = 1 corresponds to baseline (V=10 in the current study). Let D_v_, and Y_v_ denote indicators of death and cognitive impairment, respectively, by visit v. Since no participant is dead or has experienced cognitive impairment at baseline, D_1_ ≡ Y_1­_≡ 0. Similarly, let A_v_ denote cancer status at visit v, and L_v_ denote a vector of covariates (some of which may be time-varying) at visit v. If an individual dies by visit v without a history of cognitive impairment (Y_v-1_ = 0, D_v_ = 1), then all future indicators of cognitive impairment will be deterministically 0 (Y_v_=…=Y_10_ = 0), because an individual who dies without cognitive impairment, cannot experience subsequent cognitive impairment. Using this notation, we provide expressions for the different types of hazards compared. The hazard ratios (HRs) are provided below in Table S1.

Note that notation for censoring is not introduced; this is because the hazards shown below are estimands (population parameters that are targets of estimation), not estimators. We aim to estimate hazards that are not only generalizeable to the uncensored population, but are generalizeable to the population at risk irrespective of censoring. As described in the main text, we accomplish this goal using inverse probability weights for censoring, otherwise estimation using unweighted analysis would either be interpreted as explicitly conditioning on being uncensored or as relying on the assumption of noninformative censoring to estimate the hazards below.

**Cause-Specific Hazard**

The cause-specific hazard at visit v equals $\Pr\left( Y_{v}=1 \right|A_{v-1}, L_{v-1}, Y_{v-1}= D_{v}=0)$, where v>1. The risk set for cause-specific hazards includes participants with ${(Y}_{v-1}= D_{v}=0).$ Therefore, this hazard involves explicitly conditioning on survival at visit v, which is an intervening variable that occurs after the exposure ($A_{v-1})$ but before the outcome ($Y_{v}$).

**Marginal Hazard**

The marginal hazard at visit v equals $\Pr\left( Y_{v}^{\bar{d}=0}=1 \right|A_{v-1}, L_{v-1}, Y_{v-1}^{\bar{d}=0}=0)$ $=$

$\Pr\left( Y_{v}=1 \right|A_{v-1}, L_{v-1}, Y_{v-1}= D_{v}=0)\times\Pr\left( D_{v}=0 \right|A_{v-1}, L_{v-1}, Y_{v-1}=0)$, where v>1. With a slight abuse of notation from the causal inference literature,^2^ the variable $Y_{v}^{\bar{d}=0}$ denotes cognitive impairment status at visit v if death at or before visit v is eliminated as a competing risk (the formal expression is on the right-hand side of the equation). The statistical “elimination” is performed using a weighted average of the cause-specific hazard as expressed on the right-hand side of the equation. Specifically, the cause-specific hazard is weighted by the probability of survival. If death and cognitive impairment are conditionally independent, then the marginal hazard and cause-specific hazard are equal. The use of inverse probability weights to address death relaxes the assumption of conditional independence.

**Subdistribution Hazard**

The subdistribution hazard at visit v equals $\Pr\left( Y_{v}=1 \right|A_{v-1}, L_{v-1}, Y_{v-1}=0),$where v>1. Note that $(Y_{v-1}=0)$ ≡ ${(Y}_{v-1}= D_{v}=0)$ $\cup$ ${(Y}_{v-1}=0, D_{v}=1)$, where individuals with ${(Y}_{v-1}=0, D_{v}=1)$ deterministically have $Y_{v}=0$. This implies that the risk set for the subdistribution hazard includes the risk set for the cause-specific hazard as well as participants who died without cognitive impairment. Therefore, the subdistribution hazards are interpreted as hazards without eliminating the competing risk of death. Instead, death is treated as one way that participants do not develop cognitive impairment.

**Composite-Outcome Hazard**

The composite-outcome hazard at visit v equals $\Pr\left( Y_{v}=1 \cup D_{v}=1 \right|A_{v-1}, L_{v-1}, Y_{v-1}=D_{v-1}=0)$, where v>1. Notably, the risk set for the composite-outcome hazard is the same as that for the cause-specific hazard. However, the outcome differs from that for all other hazards. Specifically, the outcome is incident cognitive impairment or death.

1. **Inverse probability weight (IPW) models**

**Models for Missing Covariates**

IPWs to address missing baseline covariates were constructed from probabilities estimated by logistically regressing an indicator for complete baseline covariates on

sex, age, race, study site, physical activity, prevalent cancer history, prevalent hypertension history, prevalent diabetes history, prevalent cardiovascular disease history, and renal disease. Models included interaction terms of race with prevalent cancer history, sex, and age; sex with age and physical activity; age with study site; and prevalent cancer history with prevalent hypertension history.

**Models for Informative Censoring**

IPWs to address censored cognition status were constructed from probabilities estimated by logistically regressing an indicator for observed (non-censored) cognition status on sex, age, race, study site, physical activity, cancer history (prevalent and incident cancer history cases separated), hypertension history, diabetes history, cardiovascular disease history, renal disease, *APOE*ε4 carrier, education, smoking, alcohol intake, CES-D, baseline 3MS, and marital status among those with complete baseline covariates who were still in the risk set, which depended on the time-to-event model and its corresponding estimand (supplementary methods 1 above). Models included interaction terms of race with cancer history, hypertension history, diabetes history, cardiovascular disease history, renal disease, sex, education, and age; *APOE*ε4 carrier with study site, smoking status, and marital status, sex with age and CES-D, and cancer history with diabetes history.

**Models for Death**

IPWs for death for use with marginal hazards model were constructed from probabilities estimated by logistically regressing an indicator for being alive on the same covariates and interaction terms as we did for censoring models described above among those with complete baseline covariates who were still in the risk set.

Table S1. Hazard ratio (HR) estimands, notation, and their interpretation.

| Estimand | Notation | Interpretation |
| --- | --- | --- |
| Cause-Specific HR | $\frac{\Pr\left( Y_{v}=1 \right\vert A_{v-1}=1, L_{v-1}, Y_{v-1}= D_{v}=0)}{\Pr\left( Y_{v}=1 \right\vert A_{v-1}=0, L_{v-1}, Y_{v-1}= D_{v}=0)}$ | HR for cognitive impairment at visit v comparing those with and without a history of cancer at visit v-1 conditioned on being alive at visit v, for all v=1,…,V. |
| Marginal HR | $\frac{\Pr\left( Y_{v}=1 \right\vert A_{v-1} =1, L_{v-1}, Y_{v-1}= D_{v}=0)\times\Pr\left( D_{v}=0 \right\vert A_{v-1}=1, L_{v-1}, Y_{v-1}=0)}{\Pr\left( Y_{v}=1 \right\vert A_{v-1}=0, L_{v-1}, Y_{v-1}= D_{v}=0)\times\Pr\left( D_{v}=0 \right\vert A_{v-1}=0, L_{v-1}, Y_{v-1}=0)}$ | HR for cognitive impairment at visit v comparing those with and without a history of cancer at visit v-1 in a pseudopopulation, created through weighting, where all are alive at visit v (i.e., eliminating death as a competing risk), for all v=1,…,V. |
| Subdistribution HR | $\frac{\Pr\left( Y_{v}=1 \right\vert A_{v-1}=1, L_{v-1}, Y_{v-1}=0)}{\Pr\left( Y_{v}=1 \right\vert A_{v-1}=0, L_{v-1}, Y_{v-1}=0)}$ | HR for cognitive impairment at visit v comparing those with and without a history of cancer at visit v-1 without eliminating death as a competing risk, for all v=1,…,V. |
| Composite-Outcome HR | $\frac{\Pr\left( Y_{v}=1 \cup D_{v}=1 \right\vert A_{v-1}=1, L_{v-1}, Y_{v-1}=D_{v-1}=0)}{\Pr\left( Y_{v}=1 \cup D_{v}=1 \right\vert A_{v-1}=0, L_{v-1}, Y_{v-1}=D_{v-1}=0)}$ | HR for cognitive impairment or death at visit v comparing those with and without a history of cancer at visit v-1, for all v=1,…,V. |

Table S2. Cumulative incidence of all-cause mortality by prevalent (baseline) cancer history status.

|  | Prevalent Cancer History (n=451) | |  | No Prevalent Cancer History (n=2153) | |
| --- | --- | --- | --- | --- | --- |
| Years Since Baseline | Cumulative Incidence | 95% Confidence Interval |  | Cumulative Incidence | 95% Confidence Interval |
| 0 | 0.0% | -- |  | 0.0% | -- |
| 1 | 0.9% | 0.0%, 1.7% |  | 1.0% | 0.6%, 1.4% |
| 2 | 2.9% | 1.3%, 4.4% |  | 3.2% | 2.5%, 3.9% |
| 3 | 6.0% | 3.8%, 8.2% |  | 5.3% | 4.4%, 6.3% |
| 4 | 8.9% | 6.2%, 11.5% |  | 7.2% | 6.1%, 8.3% |
| 5 | 13.1% | 9.9%, 16.1% |  | 10.4% | 9.1%, 11.7% |
| 6 | 16.9% | 13.3%, 20.2% |  | 13.4% | 11.9%, 14.8% |
| 7 | 23.3% | 19.3%, 27.1% |  | 16.9% | 15.3%, 18.5% |
| 8 | 26.9% | 22.6%, 30.8% |  | 20.5% | 18.8%, 22.2% |
| 9 | 30.9% | 26.5%, 35.0% |  | 24.3% | 22.5%, 26.1% |

Table S3. Cumulative incidence of cognitive impairment by prevalent (baseline) cancer history status.

|  | Prevalent Cancer History (n=451) | |  | No Prevalent Cancer History (n=2153) | |
| --- | --- | --- | --- | --- | --- |
| Years Since Baseline | Cumulative Incidence | 95% Confidence Interval |  | Cumulative Incidence | 95% Confidence Interval |
| 0 | 0.0% | -- |  | 0.0% | -- |
| 1 | 0.0% | -- |  | 0.1% | 0.0%, 0.2% |
| 2 | 5.1% | 3.1%, 7.2% |  | 7.6% | 6.5%, 8.7% |
| 4 | 13.0% | 9.8%, 16.2% |  | 15.6% | 14.0%, 17.2% |
| 5 | 13.2% | 10.0%, 16.4% |  | 16.1% | 14.6%, 17.7% |
| 6 | 14.4% | 11.1%, 17.8% |  | 17.3% | 15.7%, 18.9% |
| 7 | 24.1% | 19.8%, 28.4% |  | 25.3% | 23.4%, 27.3% |
| 8 | 25.3% | 20.9%, 29.7% |  | 26.6% | 24.6%, 28.6% |
| 9 | 32.5% | 27.6%, 37.4% |  | 32.9% | 30.7%, 35.1% |

Table S4. Prevalence and cumulative incidence of cancer history.

| Years Since Baseline | Cumulative Incidence | 95% Confidence Interval |
| --- | --- | --- |
| 0 | 17.3% | 15.9%, 18.8% |
| 1 | 19.5% | 18.0%, 21.1% |
| 2 | 21.3% | 19.7%, 22.8% |
| 3 | 22.9% | 21.2%, 24.5% |
| 4 | 24.5% | 22.9%, 26.2% |
| 5 | 26.5% | 24.8%, 28.2% |
| 6 | 28.2% | 26.5%, 30.0% |
| 7 | 29.9% | 28.2%, 31.7% |
| 8 | 31.0% | 29.2%, 32.8% |
| 9 | 32.5% | 30.7%, 34.3% |

Table S5. Association of cancer history with incident cognitive impairment and all-cause mortality without inverse probability weights for censoring and missing covariates.*

| Outcome | Discrete-Time Model | HR (95% CI)† | P-value |
| --- | --- | --- | --- |
| Cognitive Impairment | Cause-Specific Hazard | 0.849 (0.723, 0.998) | 0.047 |
|  | Marginal Hazard | 0.847 (0.721, 0.995) | 0.044 |
|  | Subdistribution Hazard | 0.758 (0.642, 0.896) | 0.001 |
| Composite‡ | Cox Hazard | 1.148 (1.016, 1.297) | 0.027 |
| All-Cause Mortality | Cox Hazard | 1.809 (1.522, 2.150) | <0.001 |

*Includes N=2604 participants (all cancer history cases) and adjusts for time-varying diabetes history, hypertension history, and cardiovascular disease history; †HR: hazard ratio, CI: confidence interval; ‡Composite outcome of incident cognitive impairment or all-cause mortality.

Table S6. Association of cancer history with incident cognitive impairment and all-cause mortality without Teng Modified Mini-Mental State assessments at years 7 and 9.*

| Outcome | Discrete-Time Model | HR (95% CI)† | P-value |
| --- | --- | --- | --- |
| Cognitive Impairment | Cause-Specific Hazard | 0.818 (0.701, 0.954) | 0.011 |
|  | Marginal Hazard | 0.812 (0.696, 0.948) | 0.009 |
|  | Subdistribution Hazard | 0.758 (0.645, 0.892) | 0.008 |
| Composite‡ | Cox Hazard | 1.138 (1.007, 1.286) | 0.038 |
| All-Cause Mortality | Cox Hazard | 1.813 (1.525, 2.156) | <0.001 |

*Includes N=2604 participants (all cancer history cases) and adjusts for time-varying diabetes history, hypertension history, and cardiovascular disease history; †HR: hazard ratio, CI: confidence interval; ‡Composite outcome of incident cognitive impairment or all-cause mortality.

1. Teng EL, Chui HC. The Modified Mini-Mental State (3MS) examination. *The Journal of clinical psychiatry.* 1987;48(8):314-318.

2. Young JG, Stensrud MJ, Tchetgen Tchetgen EJ, Hernan MA. A causal framework for classical statistical estimands in failure-time settings with competing events. *Stat Med.* 2020;39(8):1199-1236.
